# A New Approach for Wounding Research: MYC2 Gene Expression and Protein Stability in Wounded *Arabidopsis* Protoplasts

**DOI:** 10.3390/plants10081518

**Published:** 2021-07-25

**Authors:** Seungmin Son, Miye Kwon, Jong Hee Im

**Affiliations:** 1 Department of Life Sciences, Korea University, 145 Anamro, Sungbuk-gu, Seoul 136701, Korea; linewind@korea.kr; 2 National Institute of Agricultural Sciences, Rural Development Administration (RDA), Jeonju 54874, Korea; 3 Jeju Biodiversity Research Institute (JBRI), Jeju Technopark (JTP), Jeju 63608, Korea; 4 Department of Horticulture, Michigan State University, East Lansing, MI 48824, USA; 5 DOE Great Lakes Bioenergy Research Center, Michigan State University, East Lansing, MI 48824, USA

**Keywords:** *Arabidopsis*, LOX2, mesophyll protoplast, MYC2, wounding

## Abstract

Wounding is a constant threat to plant survival throughout their lifespan; therefore, understanding the biological responses to wounds at the cellular level is important. The protoplast system is versatile for molecular biology, however, no wounding studies on this system have been reported. We established a new approach for wounding research using mechanically damaged *Arabidopsis* mesophyll protoplasts. Wounded protoplasts showed typical wounding responses, such as increased MPK6 kinase activity and upregulated *JAZ1* expression. We also assessed expression profiles and protein stability of the basic helix-loop-helix transcription factor MYC2 in wounded protoplasts. Promoter activity, gene expression, and protein stability of MYC2 were compromised, but recovered in the early stage of wounding. In the late stage, the promoter activity and expression of MYC2 were increased, but the protein stability was not changed. According to the results of the present study, this new cell-based approach will be of use in various molecular studies on plant wounding.

## 1. Introduction

Wounding is defined as mechanical damage that occurs frequently in plants due to biotic and abiotic stresses [1]. Plant cells are protected by mechanical barriers, such as cell walls, cuticles, and trichomes; however, such barriers are compromised during wounding, and plant cells show activation of several intracellular signaling mechanisms to heal and protect against wounding [2]. Wounding generates damage-associated molecular patterns and activates MPK6 [3,4,5]. In addition, the expression of numerous genes associated with phytohormones, oxidative stress, dehydration stress, and heat-shock proteins is rapidly upregulated during wounding [6,7,8], and protein turnover, transport processes, metabolism modulation, and gene expression reprogramming occur [9]. 

Jasmonate (JA) is a major immune phytohormone that accumulates after wounding [10]. In *Arabidopsis*, the basic helix-loop-helix leu zipper transcription factor MYC2 is a major regulator of the JA signaling pathway and response [11]. Further, MYC2 is involved in various phytohormone crosstalk and several signaling pathways [12,13,14].

Continuous JA signaling is harmful and adversely affects plant growth and development [15]. The JA master regulator MYC2 is a short-lived protein, and its transcriptional activity is regulated by numerous mechanisms [16,17,18]. MYC2 transcriptional activity and protein stability require tight regulation to optimize plant fitness [19]. In the absence of JA signaling, MYC2 is repressed by a complex consisting of the JASMONATE-ZIM domain (JAZ), TOPLESS, and NOVEL INTERACTOR OF JAZ proteins [20,21,22]. In the presence of JA signaling, MYC2 is derepressed through SCF^COI1^-dependent degradation of JAZ repressors, and forms a transcriptional activation complex with MEDIATOR25 [23,24,25]. Thus, JA-triggered activation of MYC2 regulates the transcription of JA-responsive genes, including *JAZ*s and *LOX2* [26].

The plant protoplast system has been used as a versatile and powerful complex for cell-based experiments in many plant species [27,28,29]. The highly efficient protoplast transient expression systems have greatly contributed to the development of various fields of botany, including subcellular localization, protein-protein interaction, transport, signal transduction, and metabolic pathways [30,31,32,33,34,35]. In particular, transient expression in *Arabidopsis* mesophyll protoplasts has facilitated advancements in plant research. A recent study reconstituted JA signaling in *Arabidopsis* protoplasts and confirmed that the protoplast is an invaluable system for functional analysis of signaling components involved in the JA signaling pathway [36]. 

The protoplast system was previously used to study the effects of various environmental stresses [37]; however, cell-based wounding response methods have not been explored. Here, we analyzed wounding responses in mechanically damaged *Arabidopsis* mesophyll protoplasts. We further determined MYC2 transcriptional activity and protein stability in these protoplasts. This cell-based study shows wounding response in protoplast cells.

## 2. Results

### 2.1. Mechanical Wounding of Arabidopsis Mesophyll Protoplasts

**Figure 1:**
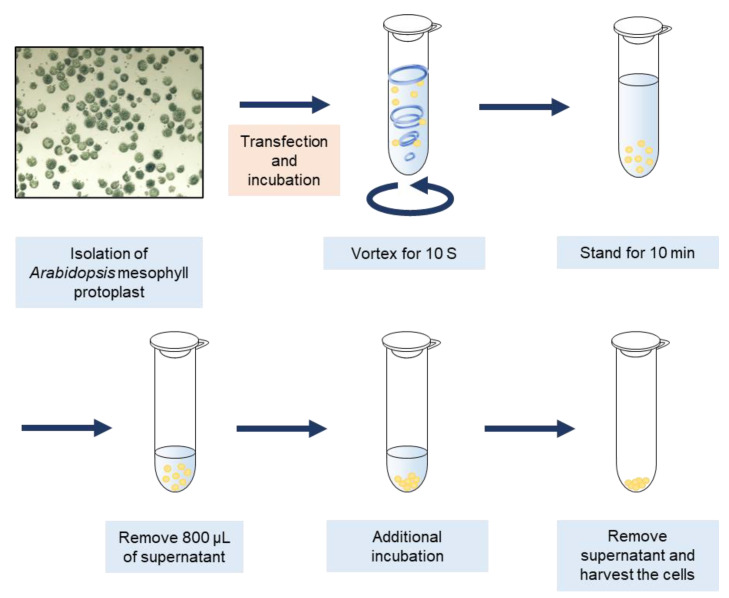
Schematic representation of vortex-induced wounding in *Arabidopsis* mesophyll protoplasts. *Arabidopsis* mesophyll protoplast (AMPs) were isolated and transfected with transiently expressing DNA, followed by incubation for desired time. Wounding was induced by vigorous vortexing for 10 s, followed by incubation for 10 min. The supernatant was removed to reduce hypoxia, followed by further incubation. Protoplasts were harvested after the complete removal of the supernatant.

We first isolated *Arabidopsis* mesophyll protoplasts (AMPs) and transfected DNA using a previously described method [28,38]. Subsequently, the cells were placed in 1.5 mL tubes at a volume of approximately 1 mL. To induce mechanical damage in AMPs, the transfected cells were vigorously vortexed for 10 s and were allowed to stand for 10 min to precipitate at the bottom of the tube. Thereafter, 800 μL supernatant was removed from the tube to reduce hypoxia, and the cells were then incubated. After incubation, the supernatant was completely removed, and the cells were harvested (Figure 1).

### 2.2. Vortex-Induced Damage Generated Typical Wounding Responses in Protoplasts

To analyze whether vortex-induced damage would generate wounding responses in AMPs, we distinguished three types of protoplasts based on their shapes: normal shaped cells (NSC), weakly wounded cells (WWC), and severely wounded cells (SWC). NSC had a round shape, and chloroplasts were evenly separated in all cell areas. WWC had a rough cell surface and, even though the cell surfaces were round, chloroplasts were not equally distributed. SWC showed the complete loss of the round shape, and chloroplasts were localized on one side (Figure S1).

We compared the proportions of cell types under normal conditions and after wounding. After transfection of 200 μL of AMPs (4–5 × 10^4^ protoplasts in 200 μL) with 40 μg of empty vector, the cells were harvested and wounded through vigorous vortexing at 3200 rpm for 5, 10, 15, and 20 s. In the controls, NSCs accounted for 86.01% ± 3.52%, WWCs accounted for 7.86% ± 0.98%, and SWCs accounted for 6.12% ± 1.38% of the cells (Figure S2A). However, the composition was significantly altered following wounding. In cells vortexed for 10 s, approximately 70% of the cells showed altered shapes, and cells vortexed for 15 s were markedly disrupted (Figure S2A,B). 

**Figure 2:**
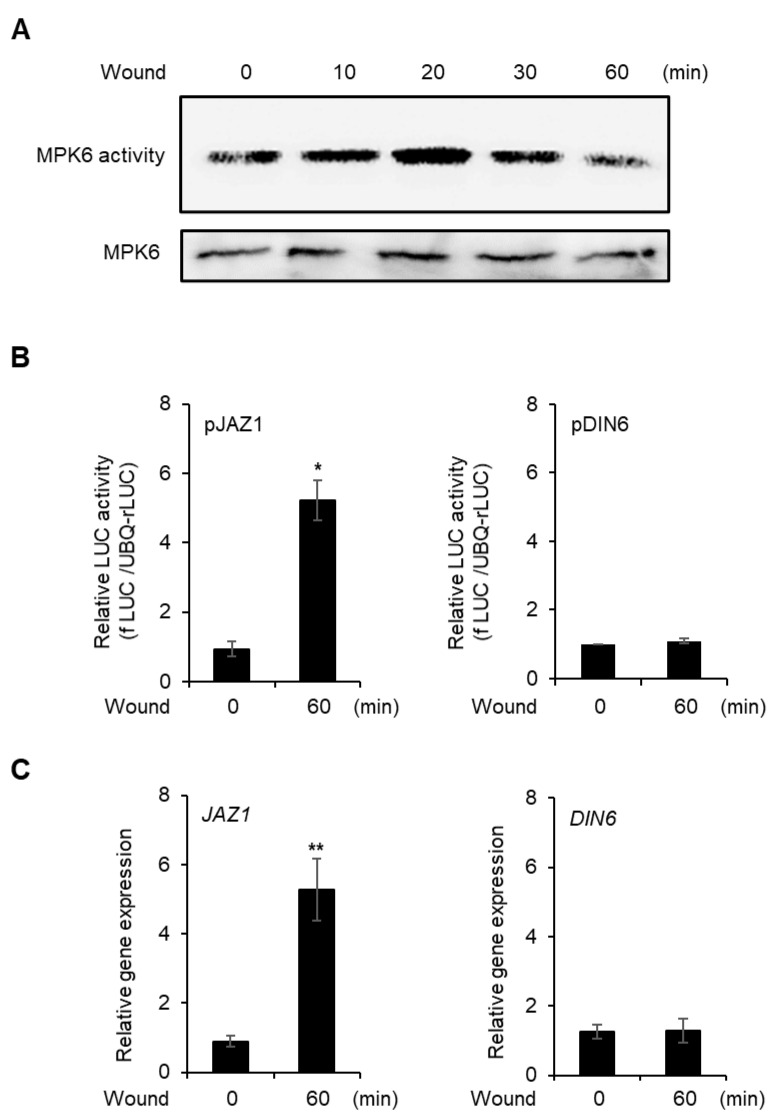
Response of vortex-induced wounding. (**A**) in vitro kinase activity of MPK6. After MPK6 was expressed in protoplast, vortex-induced wounding was carried out and time-dependent MPK6 activity was determined. Myelin basic protein was used as kinase substrate. The (**B**) promoter activities of *JAZ1* and *DIN6* after 60 min of wounding in AMPs. The promoter of *JAZ1* and *DIN6* was transfected to AMPs and incubated for 6 h. The AMPs were wounded and additionally incubated for 60 min and promoter activities were analyzed. Values are means ± SE of three repeats: * *p* < 0.01. The (**C**) gene expression of *JAZ1* and *DIN6* in wounded protoplasts. After isolation of AMPs, the protoplasts were incubated for 4 h without transfection for stabilization and wounded by vortex and incubated for 60 min. Total RNA was isolated from the AMPs and RT-qPCR was carried out. Values are means ± SE of three repeats: ** *p* < 0.001.

To verify whether vortex-induced damage would induce a wounding response in the cells, we analyzed MPK6 kinase activity because MPK6 is activated by wounding [39]. After MPK6 was expressed with *35S* promoter in AMPs, the cells were vortexed, and MPK6 activity was measured for 60 min. MPK6 activity peaked 20 min after wounding and decreased thereafter (Figure 2A and Figure S3).

We also analyzed the promoter activity and gene expression of *JAZ1* for 60 min after vortexing because *JAZ1* expression is rapidly increased under wounding stress [40]. Promoter activity was not changed in the non-wounded protoplasts, but the promoter activity and expression of *JAZ1* were significantly increased following wounding (Figure 2B and Figure S4A); however, the hypoxia marker gene, *DIN6*, did not change between normal condition and wounding treatment (Figure 2C and Figure S4B). The data suggest that vortex-induced mechanical damage to protoplasts exhibits typical responses of wounding stress.

### 2.3. Gene Expression and Protein Stability of MYC2 Are Compromised and Recovered in Early Stage of Wounding

**Figure 3:**
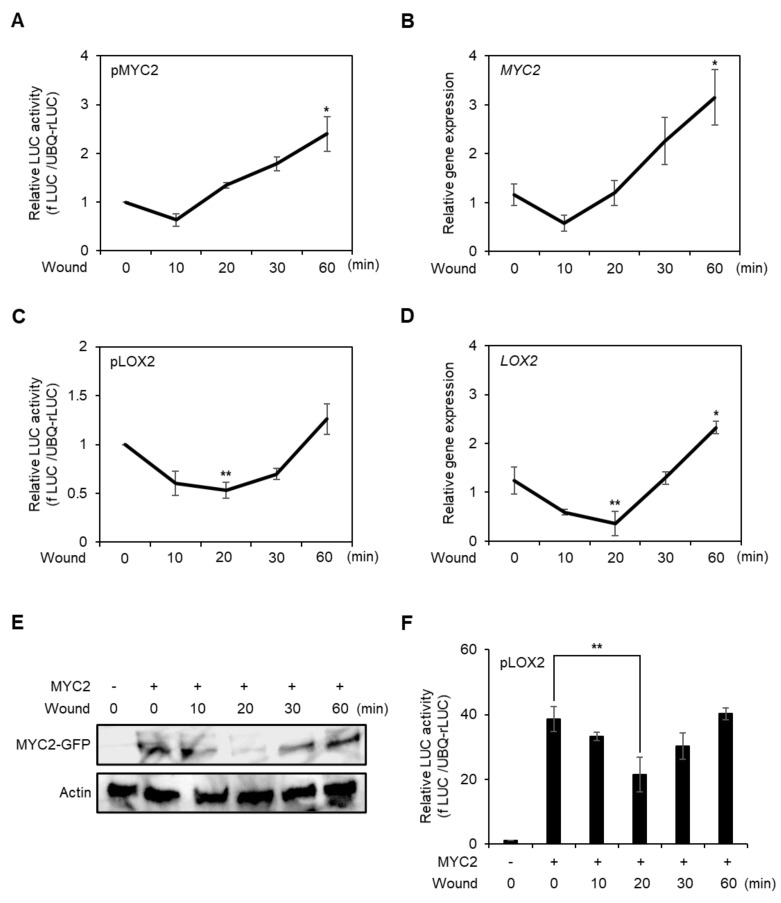
Analysis of promoter activity, gene expression, and protein stability of MYC2 during the early stage of wounding in protoplasts. Promoter activity of *MYC2* (**A**) and *LOX2* (**C**) in the early stage of wounded protoplast. *fLUC*-conjugated *MYC2* and *LOX2* promoters were respectively transfected to AMPs and incubated for 6 h. Vortex-induced wounding was performed, and the promoter activity was determined in a time-dependent manner. *UBQ*-*rLUC* was used as an expression control. Values are means ± SE of three repeats: * *p* < 0.01 and ** *p* < 0.001. AMPs were incubated for 4 h without transfection, wounded by vortex, and incubated for the designated time points. Total RNA was isolated from the AMPs and RT-qPCR was carried out with gene-specific primers of *MYC2* (**B**) and *LOX2* (**D**). *Actin2* was used as an expression control. Values are means ± SE of three repeats: * *p* < 0.01 and ** *p* < 0.001. (**E**) Analysis of MYC2 protein stability in wounded protoplasts. *35S* promoter-driven C-terminal *GFP*-conjugated *MYC2* was transfected and incubated for 10 h. The protoplasts were wounded and incubated for designated time points and harvested. MYC2 proteins were detected using an anti-GFP antibody. Actin was used as a loading control. (**F**) Transient promoter activity of *LOX2* with MYC2 effector in wounded protoplast. *fLUC*-conjugated *LOX2* was expressed with or without MYC2 effector and incubated for 60 min. The *LOX2* promoter activity was determined in a time-dependent manner. *UBQ*-*rLUC* was used as an expression control. Values are means ± SE of three repeats: ** *p* < 0.001.

JA is a major hormone of the wounding response, and MYC2 is a master regulator of JA signaling [12]. Therefore, we determined the MYC2 promoter activity, gene expression, and protein stability during the early stage of wounding. To analyze the *MYC2* promoter activity, we transfected the *fLUC* conjugated *MYC2* promoter to the AMPs and incubated them for 6 h, then wounded the AMPs by vortexing and incubated the cells for 60 min. *MYC2* promoter activity decreased 10 min after wounding, but recovered quickly and increased for 60 min after the treatment (Figure 3A). This pattern was correlated with *MYC2* gene expression (Figure 3B).

To analyze the transcriptional activity of MYC2 in the wounded protoplasts, we measured *LOX2* promoter activity and gene expression caused by direct targeting of MYC2 [41]. The *LOX2* promoter activity and gene expression patterns were similar to those of MYC2, but recovery took longer (Figure 3C,D). Therefore, we investigated MYC2 protein stability in wounded protoplasts. C-terminal *GFP*-conjugated *35S* promoter-driven *MYC2* DNA was transfected into AMPs and then the protoplasts were wounded for 10 s. The MYC2 protein stability was determined for 60 min. Protein stability was compromised until 20 min after wounding, but increased subsequently (Figure 3E and Figure S5A). MYC2 protein stability was correlated with MYC2-induced *LOX2* promoter activity (Figure 3F). The data indicated that MYC2 expression and protein stability were compromised in wounded protoplasts, and then recovered in the early stage. 

### 2.4. Gene Expression of MYC2 Is Increased with Stable Protein Expression at Late Stage of Wounding

**Figure 4:**
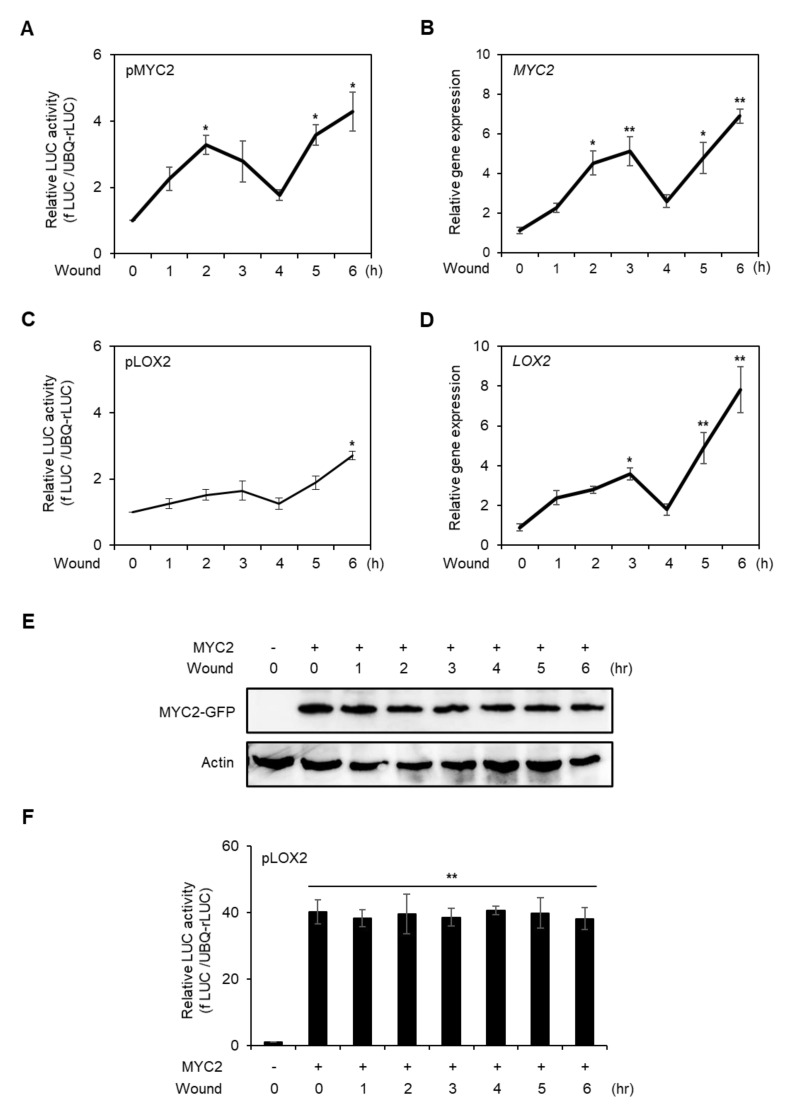
Analysis of promoter activity, gene expression, and protein stability of MYC2 in wounded protoplast at the late stage of wounding. Promoter activities of *MYC2* (**A**) and *LOX2* (**C**) in the late stage of wounded protoplast. *fLUC*-conjugated *MYC2* and *LOX2* promoters were respectively transfected to AMPs and incubated for 6 h. Vortex-induced wounding was generated in AMPS and incubated for 6 h. The promoter activities were determined in a time-dependent manner. *UBQ*-*rLUC* was used as an expression control. Values are means ± SE of three repeats: * *p* < 0.01. AMPs were incubated for 4 h without transfection and wounded by vortex and incubated for the designated time points. Total RNA was isolated from the AMPs and RT-qPCR was carried out with gene-specific primers of *MYC2* (**B**) and *LOX2* (**D**). *Actin2* was used as an expression control. Values are means ± SE of three repeats: * *p* < 0.01 and ** *p* < 0.001. (**E**) Analysis of MYC2 protein stability in wounded protoplasts. *35S* promoter-driven C-terminal *GFP*-conjugated *MYC2* was transfected and incubated for 10 h. The protoplasts were wounded and incubated for designated time points and harvested. MYC2 proteins were detected using an anti-GFP antibody. Actin was used as a loading control. (**F**) Transient promoter activity of *LOX2* with MYC2 effector in wounded protoplast. *fLUC*-conjugated *LOX2* was expressed with or without MYC2 effector and incubated for 6 h. The *LOX2* promoter activity was determined in a time-dependent manner. *UBQ*-*rLUC* was used as an expression control. Values are means ± SE of three repeats: ** *p* < 0.001.

We further analyzed MYC2 expression and protein stability over a longer period in wounded protoplasts. The promoter activity and gene expression of *MYC2* fluctuated, but showed increasing trends until 6 h after wounding (Figure 4A,B). The *LOX2* promoter activity and gene expression patterns were similar to those of *MYC2* (Figure 4C,D), suggesting that MYC2 protein stability is not altered in the late stage of wounding.

To verify the above possibility, we expressed C-terminal *GFP*-conjugated *35S* promoter-driven *MYC2*, performed wounding treatments, and then measured MYC2 protein stability for 6 h in AMPs. As shown in Figure 4E and Figure S5B, the MYC2 protein stability did not change during the 6 h after wounding. To further verify the stability of MYC2 protein, *LOX2* promoter activity was measured. The *LOX2* promoter activity increased under MYC2 co-expression, but did not change until after 6 h of wounding. The results indicated that MYC2 expression increased in the late stage of wounded protoplasts without post-translational modification.

## 3. Discussion

The protoplast system is versatile and has been used for various abiotic stresses but not applied for wounding study [40,42,43]. Here, we established a novel method based on *Arabidopsis* mesophyll protoplasts (AMPs) for analysis of wounding response. We induced mechanical damage to AMPs through vigorous vortexing, which caused damage to all protoplasts and altered the shapes of approximately 66% of the AMPs. Furthermore, wounding increased MPK6 activity and *JAZ1* expression (Figure 2). These effects were typical wounding-induced responses. Consequently, the vortex-induced mechanical damage generates a wounding response in AMPs.

JA is an important hormone of the wounding response, and MYC2 is a key regulator of JA signaling. Therefore, we analyzed MYC2 expression profiles and protein stability in wounded protoplasts. During the early wounding response, *MYC2* expression in *Arabidopsis* leaves was significantly increased at 30 min and 1 h, and it was decreased 3 h after wounding [44]. However, earlier responses were not reported. The *MYC2* promoter activity and expression were reduced 10 min after wounding and exhibited rapid recovery (Figure 3A,B). This was a so-far unknown response in wounded cells.

We further analyzed *MYC2* gene expression and promoter activity in wounded protoplasts with *LOX2*. *LOX2* promoter activity and gene expression decreased and recovered; however, recovery occurred later than that of *MYC2* (Figure 3C,D), indicating that the MYC2 protein is not stable in wounded protoplasts. To verify this possibility, we determined the stability of the MYC2 protein in wounded protoplasts. MYC2 protein was degraded at 20 min and then recovered (Figure 3E,F). This post-translational modification may be regulated by kinases as numerous kinases are activated during wounding [45,46,47]. This means that MYC2 may be negatively regulated by one of the activated kinases during early wounding.

Subsequently, we analyzed MYC2 expression and protein stability during the late stage of protoplast wounding. We limited the analysis time to 6 h after wounding because AMPs turned unstable 24 h after isolation (Figure S4). *MYC2* promoter activity and gene expression increased, and *LOX2* exhibited a similar pattern (Figure 4A–D), suggesting that MYC2 is stable in the late stage of wounding, as verified using protein blotting of MYC2 (Figure 4E) and by assessing *LOX2* promoter activity with MYC2 effector co-expression (Figure 4F). The results indicated that MYC2 expression increased without post-translational modification in the late stage of the wounding.

Wounding treatment of leaves may be associated with a time gap between the first and last treatment, which can be reduced using this protoplast system with vortex-induced wounding. This is an advantage of using vortex-induced wounding.

The novel experimental model outlined in the present study displays the responses of wounded cells, which could be improved by the adoption of a single-cell-based multi-omics platform.

## 4. Materials and Methods

### 4.1. Plant Material and Growth Conditions

*Arabidopsis thaliana* Col-0 plants were used. For protoplast generation, plants were grown in Professional Growth Mix soil (Sun Gro, Agawam, MA, USA) for 23–25 days with programmable light (12 h, 50–70 μE) and dark (12 h) conditions at 23 °C. Humidity was adjusted to 40–60%.

### 4.2. Protoplast Isolation and Polyethylene Glycol (PEG) Transfection

Protoplast isolation and polyethylene glycol (PEG)-mediated transfection was performed as described previously [28,38], with slight modifications. Briefly, 24-day-old plants that were grown in soil were cut into small pieces using a razor blade and incubated for 4 h in an enzyme solution (20 mM MES-KOH [pH 5.7], 1.5% cellulase R10, 0.4% macerozyme R10, 0.4 M mannitol, 20 mM KCl, and 10 mM CaCl_2_). After centrifugation, 4–6 × 10^4^ protoplasts were resuspended in a 200 µL MMG solution (4 mM MES-KOH [pH 5.7], 0.4 M mannitol, and 15 mM MgCl_2_). A total of 20 µg of constructs were mixed well with 200 µL of protoplasts and a PEG solution (40% PEG4000, 0.2 M mannitol, and 100 mM CaCl_2_). After 4 min of incubation, a WI solution (4 mM MES-KOH [pH 5.7], 0.5M mannitol, and 20 mM KCl) was added to the sample. The protoplasts were incubated and harvested. 

### 4.3. In Vitro Kinase Assay

For the kinase assay, *MPK6* was inserted into the *HBT* promoter and the *NOS* terminator in the transient expression vector pHBT-HA. The construct was transfected into mesophyll protoplasts and incubated for the indicated times. The cells were lysed, and the protein extracts were incubated with an anti-HA antibody and the additional adding of A-agarose beads. After bead washing, the immune complex kinase assay of MPK6 was performed as described previously [48]. Briefly, purified MPK6-HA was mixed with 3 µg of myelin basic protein in a kinase reaction buffer (50 mM Tris-HCl [pH 7.5], 10 mM MgCl_2_, 1 mM DTT, and 50 μM [γ-32P] ATP) for 30 min at room temperature. The reaction was stopped by a SDS loading buffer, and an equal volume of each sample was loaded into a 10% SDS-PAGE gel. After the separating, phosphorylation was detected with a phosphor-image analyzer (FLA-7000, Fujifilm, Japan). The experiment was independently conducted at least three times, and representative data are shown.

### 4.4. Transient Promoter Assay

The protoplast transient promoter assay was performed as described previously [27]. To generate an effector construct for transient expression in protoplasts, *MYC2* was cloned into the pHBT-GFP vector. To generate the reporter plasmids, 1 kb upstream promoter regions of *DIN6*, *LOX2*, *JAZ1*, and *MYC2* were cloned into the firefly luciferase vector. For luciferase assays, 8 µg of reporter plasmid and 1 µg of *pUBQ-rLUC* [49] were transfected into protoplasts and incubated at 23 °C. After incubation, reporter activities were measured using a dual luciferase assay system (Promega, Madison USA). 

### 4.5. RNA Extraction and RT-qPCR

The isolated protoplasts were incubated at room temperature for stabilization, followed by vortex-induced wounding, and then incubated for the designated period. The total RNA was extracted from the protoplasts using a TRIzol reagent (Invitrogen, Waltham USA), and 200 ng of total RNA was used for the first-strand cDNA synthesis using Superscript III reverse transcriptase (Invitrogen, Waltham USA). A quantitative real-time polymerase chain reaction (RT-qPCR) was performed using specific primers (Table S1) and conducted on the MyiQ Real-Time PCR System (Bio-Rad, Hercules USA) using the SYBR Green Master Mix (Bio-Rad, Hercules USA) under the following conditions: 40 cycles of denaturation at 95 °C for 10 s, annealing at 58 °C for 15 s, and extension at 72 °C for 30 s. The gene expression was quantified using the comparative Ct method. *Actin* was used as a calibration control to determine the expression of genes. The experiment was independently conducted at least three times.

### 4.6. Protein Blot Analysis

The total protein was extracted from the protoplasts using an extraction buffer (50 mM Tris-Base, 150 mM NaCl, 10 mM NaF, 10 mM Na_3_Vo_4_, 1x Complete, and 0.2% Triton X-100). The proteins were separated in 10% SDS-PAGE and transferred to polyvinylidene difluoride membranes. For immunoblotting, the primary antibodies anti-HA (Roche), anti-GFP (Abcam), and anti-ACT (Agrisera) were used (1:1000). Next, an HRP-conjugated secondary antibody (Abcam) was added (1:10,000). The signal was detected using an IR-image detector Odyssey (LI-COR, Lincoln USA). 

### 4.7. Statistical Analyses

Luciferase assays and RT-qPCRs were independently conducted at least three times, and differences were tested using a t-test in GraphPad Prism 8.0 (GraphPad Software, San Diego, CA, USA).

## Data Availability

The original data are provided in the manuscript.

## Supplementary Materials

The following are available online at https://www.mdpi.com/article/10.3390/plants10081518/s1, Figure S1: Three different cell types in wounded protoplasts. Figure S2: Proportion of protoplast types and rate of protoplast disruption following vortexing time. Figure S3: Statistical analysis of MPK6 activity in wounded protoplasts. Figure S4: Promoter activities of *JAZ1* and *DIN6* in normal condition protoplast. Figure S5: Statistical analysis of MYC2 protein stability in wounded protoplasts., Table S1: List of primers of this study.
