# Supplementary material for: A New Approach for Wounding Research: MYC2 Gene Expression and Protein Stability in Wounded Arabidopsis Protoplasts
Source: Plants (Basel). 2021 Jul 25;10(8):1518. doi: 10.3390/plants10081518 (PMC8399638; doi:10.3390/plants10081518)
Supplement: Supplementary file 1 [file plants-10-01518-s001.zip › plants-1263391-supplementary.pdf]

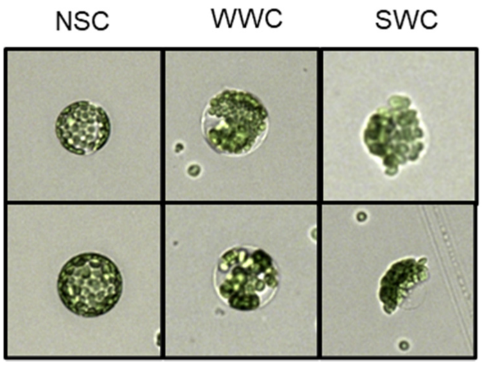

Figure S1. Three different cell types in wounded protoplasts. normal shape cell (NSC), weakly wounded cell (WWC), and severely wounded cell (SWC).

A

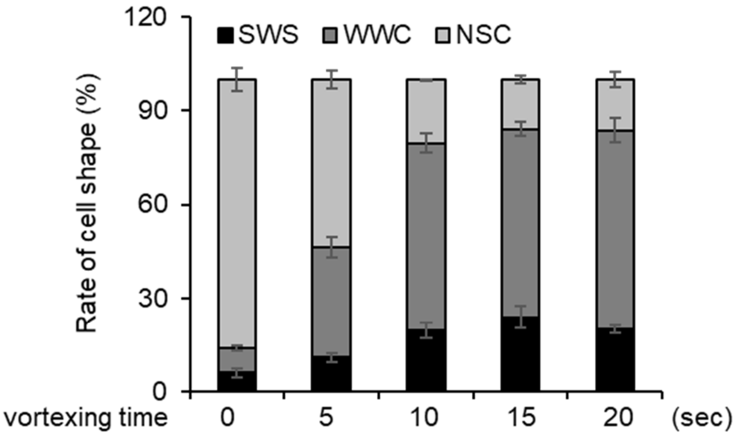

B

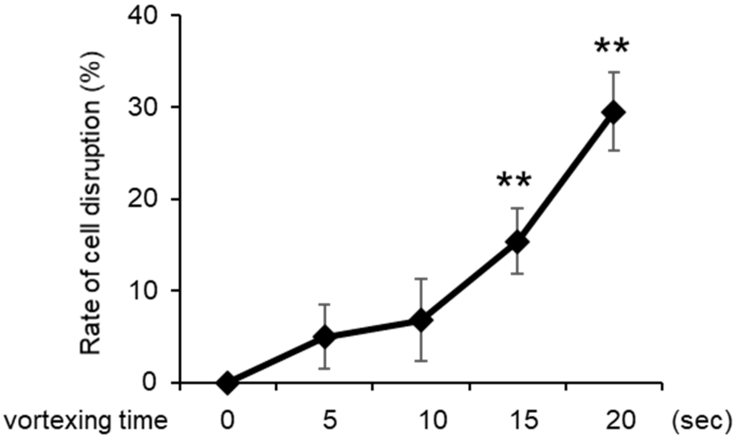

Figure S2. The proportion of protoplast types and rate of protoplast disruption following vortexing time. (A) The proportion of cell types in wounded protoplasts. After isolation of *Arabidopsis* mesophyll protoplasts (AMPs), the cells were incubated for 4 h and vortexed with 3200 rpm for designated times. Then, the shape of cells was analyzed with three different types (n=200). SWC: severely wound-ed cell; WWC: weakly wounded cell; NSC: Normal shape cell. (B) Rate of disrupted protoplasts after vortex-induced wounding. Isolated AMPs were vortexed with 3200 rpm for designated times and the total number of protoplasts were analyzed before and after wounding with hemocytometer according to the following equation:

$$Rate\ of\ cell\ disruption\ (\%) = \frac{Total\ number\ of\ cells\ ater\ wounding}{Total\ number\ of\ cells\ before\ wounding} \times 100$$

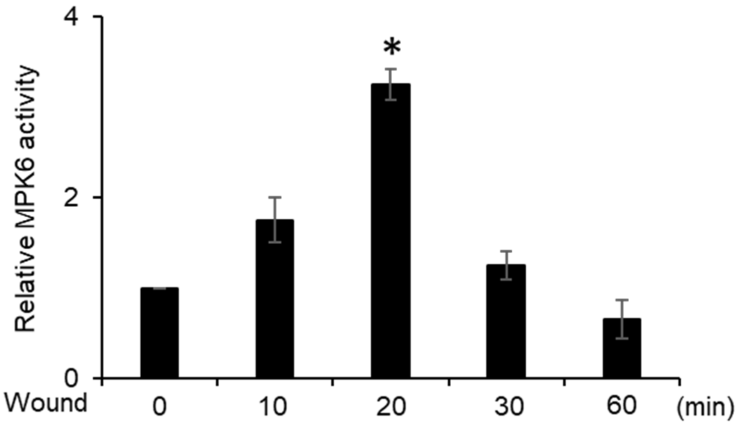

Figure S3. Statistical analysis of MPK6 activity in wounded protoplasts. *35S* promoter-driven C-terminal *HA* conjugated *MPK6* was expressed in AMPs and incubated for 6 h, then vortex-induced wounding was carried out and time-dependent MPK6 activity was determined with a scintillation counter. Values are means ± SE of three repeats: * $P < 0.01$.

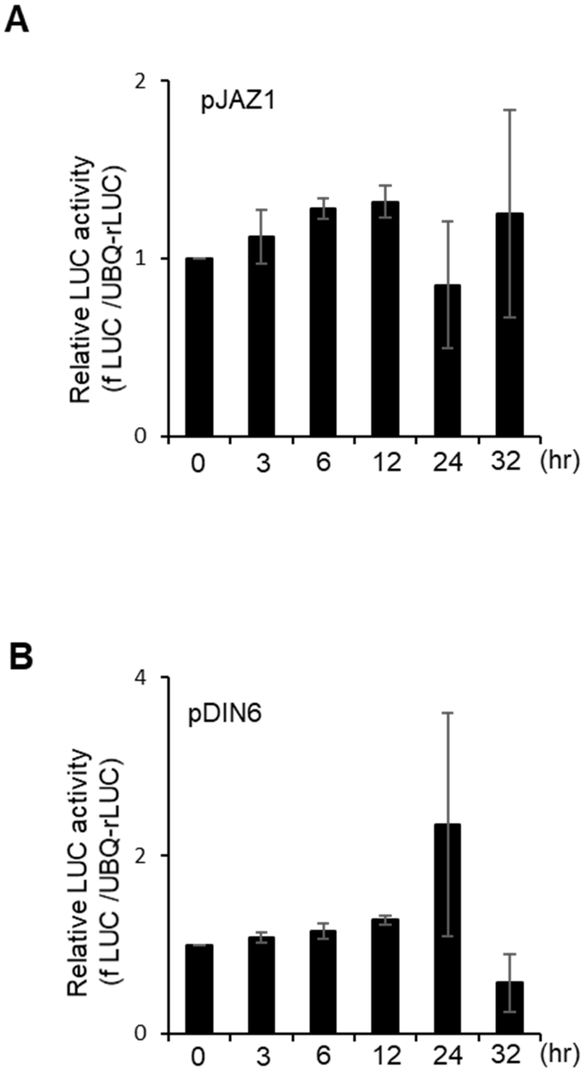

Figure S4. Promoter activities of *JAZ1* and *DIN6* in normal condition protoplast. *fLUC*-conjugated *JAZ1* (A) and *DIN6* (B) promoters were respectively transfected to AMPs and incubated for 6 h, and additionally incubated for designated time points. The promoter activity was determined in a time-dependent manner. *UBQ-rLUC* was used as an expression control.

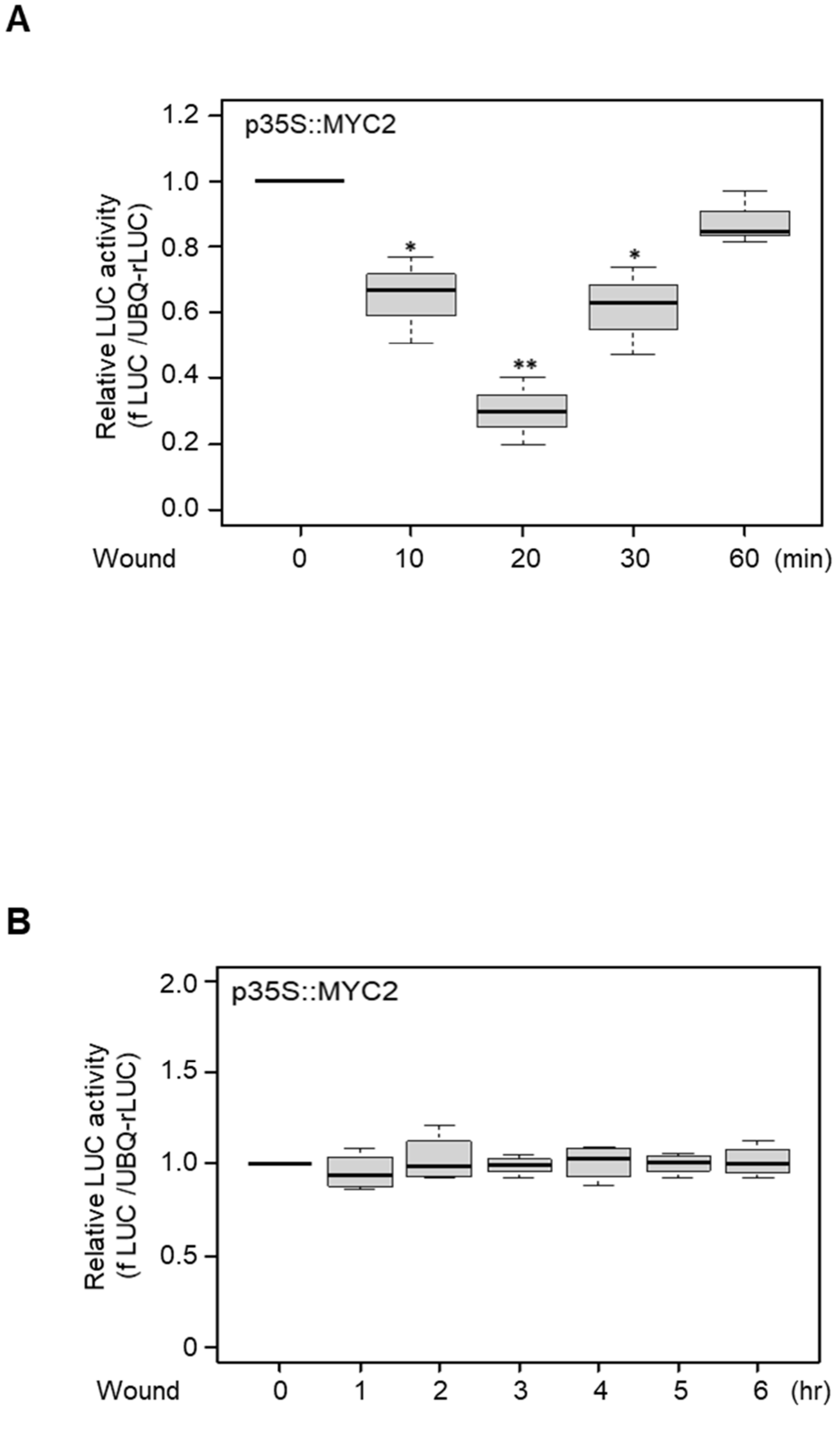

Figure S5. Statistical analysis of MYC2 protein stability in wounded protoplasts. (A) Box plot of MYC2 protein stability in the early stage of wounded protoplasts. *35S* promoter-driven C-terminal *fLUC* conjugated *MYC2* was transfected and incubated for 10 h. Vortex-induced wounding was generated in protoplasts and was harvested after incubation for designated time points. MYC2 protein stabilities were detected using a dual-luciferase system. *pUBQ-rLUC* was used as control. Values are means ± SE of three repeats: ** $P < 0.001$, * $P < 0.01$. (B) Box plot of MYC2 protein stability in the late stage of wounded protoplasts. *35S* promoter-driven C-terminal *fLUC* conjugated *MYC2* was transfected and incubated for 10 h. Vortex-induced wounding was generated in protoplasts and was harvested after incubation for designated time points. MYC2 protein stabilities were detected using a dual-luciferase system. *pUBQ-rLUC* was used as control.

Table S1. List of primers of this study.

Gene	Locus number	Primer sequence	Experiments
MPK6	AT2G43790	CGGGATCCATGGACGGTGGTTCAGG	Gene cloning
		GAAGGCCTTTGCTGATATTCTGGATTGA	
JAZ1	AT1G19180	TCTACGCCGGGCAAGTGATT	qPCR
		CTTCACTTCACCGGTTCTTGGAG	
	AT1G19180	GGATCCCTACTTTGAGAAAGCAGCTG	Promoter cloning
		CCATGGGATAAAAAAAAACAGCACCAAA	
DIN6	AT3G47340	TTCACCTTTCGGCCTACGAT	qPCR
		ATCGGCATGTTGTCAATTGC	
	AT3G47340	GGATCCATTGGGTCTTGCTGCCTC	Promoter cloning
		CCATGGCTTCTTAGTCTTTACATT	
MYC2	AT1G32640	CGACGGCGGAGCTGGAGATTTAT	qPCR
		GATTCGGGTTTTCGGTTATTGTGC	
	AT1G32640	GGATCCCATGTTCAAAAGTCGACTC	Promoter cloning
		CCATGGTTTTATAATGTTATTTTAAAATAT	
	AT1G32640	CATGCCATGGATGACTGATTACCGGCTA	Gene cloning
		GAAGGCCTACCGATTTTTGAAATCAAAC	
LOX2	AT3G45140	GCAACGCTACGGGGGAGAG	qPCR
		ACTGGGGCATCAAACTGGAGAAT	
	AT3G45140	GGATCCCTGATCAGATCCTGGACCA	Promoter cloning
		CCATGGGGTACTCTACGACGTTAAT	
